# Benzimidazole-Regulated 1D 4-Fluorosalicylic Acid MOF Composite Material Design and Its Application in Glucose Sensing

**DOI:** 10.3390/molecules31173096

**Published:** 2026-09-03

**Authors:** Haixia Wu, Dianheng Yu, Jinliang Hu, Fang Wang, Songtao Zhang, Kailu Guo, Huan Pang

**Affiliations:** 1 Henan Key Laboratory of Function-Oriented Porous Materials, College of Chemistry and Chemical Engineering, Luoyang Normal University, Luoyang 471934, China; haixiawu@lynu.edu.cn; 2 Testing Center, School of Chemistry and Materials, Yangzhou University, Yangzhou 225009, China; 221002134@stu.yzu.edu.cn (D.Y.); hujl0330@126.com (J.H.); wf393231106@126.com (F.W.); 3 Yangzhou Key Laboratory of Smart Materials and Clean Energy, Interdisciplinary Research Center for Advanced Energy, Yangzhou 225002, China

**Keywords:** metal–organic frameworks, one-dimensional nanostructure composites, nickel-based materials, electrochemical sensor, glucose

## Abstract

Metal–organic frameworks (MOFs) have significant potential in electrochemical sensors, but the guest molecules and residual solvents in the pores often block the active sites and limit the reaction kinetics. One-dimensional nanostructures can provide direct conduction pathways and shorten ion diffusion distances, thereby enhancing electron transport and electrode contact. Meanwhile, fluorine-incorporated MOF materials leverage the high electronegativity of fluorine to substitute oxygen, suppress oxidation to widen the voltage window, and improve stability through enhanced hydrophobicity. In this work, 4-fluorosalicylic acid (4FSA) was used as the ligand and benzimidazole (Bim) was introduced to adjust the coordination environment, and one-dimensional Bim4FSA-MOF nanorods were successfully constructed. While the guest molecules were largely removed, the nickel sites were thereby activated and the pore size was enlarged. Due to the synergistic effect of one-dimensional nanostructure-promoted electron transport and the Ni(OH)_2_/NiOOH dynamic active center, the B-250 composite exhibited excellent performance in a glucose electrochemical sensor. The optimized sensor delivered a detection limit of 0.022 μM and a detection time of 0.9 s, along with a sensitivity value of 2986.45 μA mM^−1^ cm^−2^, which provides a new strategy for the design of efficient MOF-based electrochemical sensor interface.

## 1. Introduction

Precise and rapid glucose detection is critical in clinical diagnostics, especially for the management of diabetes mellitus [1,2], a chronic metabolic disorder affecting over 500 million people globally. Deviations in blood glucose levels can lead to severe complications [3,4], including cardiovascular disease [5], nephropathy, and neuropathy, underscoring the critical need for continuous and accurate glucose monitoring [6,7]. While conventional enzymatic electrochemical sensors exhibit superior selectivity, they are frequently hampered by inherent instability, intricate immobilization protocols, and susceptibility to environmental variables like pH and temperature. Consequently, the advancement of robust, non-enzymatic glucose sensors utilizing advanced functional nanomaterials has become a significant research avenue [8,9], targeting enhanced sensitivity, reduced detection limits, and accelerated response times for point-of-care and wearable technologies [10].

Among the diverse array of electrode materials, metal–organic frameworks (MOFs) have garnered significant interest due to their ultra-high specific surface area, tunable porosity, and structural diversity [11,12]. Specifically, nickel-based MOFs (Ni-MOFs) are particularly attractive for non-enzymatic glucose sensing [13], capitalizing on the well-documented Ni^2+^/Ni^3+^ (NiOOH) redox couple that can effectively catalyze glucose oxidation in alkaline media [14]. Nevertheless, the practical deployment of pristine Ni-MOFs is frequently hampered by limited electrical conductivity and, more critically, by guest molecules and residual solvents confined within their pores [15,16]. These occlusions block catalytically active metal sites and impede glucose diffusion to the electrode surface [17], yielding suboptimal sensitivity, elevated detection limits, and sluggish response kinetics [18].

To overcome these inherent limitations, innovative strategies for modulating the physicochemical properties of Ni-MOFs are required [19,20,21]. In this work, we report a rational design approach involving the construction of a one-dimensional (1D) Ni-MOF using 4-fluorosalicylic acid (4FSA) as the primary ligand, followed by the strategic introduction of benzimidazole (Bim) to fine-tune the coordination environment, yielding a well-defined Bim4FSA-MOF-3 precursor. A subsequent gentle thermal treatment was applied to selectively remove the pore-occluding species without compromising the framework’s structural integrity. The process is proposed to expose the nickel sites and enlarge the pore channels, thereby facilitating rapid mass transport. The resulting optimized material, denoted B-250, synergistically combines the benefits of a 1D nanostructure for accelerated electron transfer with the dynamic generation of Ni(OH)_2_/NiOOH active centers during electrocatalysis [22]. When applied as a non-enzymatic glucose sensor, the B-250-modified electrode exhibits outstanding analytical performance, achieving a high sensitivity of 2986.45 μA mM^−1^ cm^−2^, an extremely low detection limit of 0.022 μM, and a remarkably fast response time of 0.9 s. This work presents a compelling strategy for designing high-performance MOF-based sensing interfaces by addressing the critical issue of pore accessibility and active site exposure.

## 2. Results

### 2.1. Characterization

**Figure 1:**
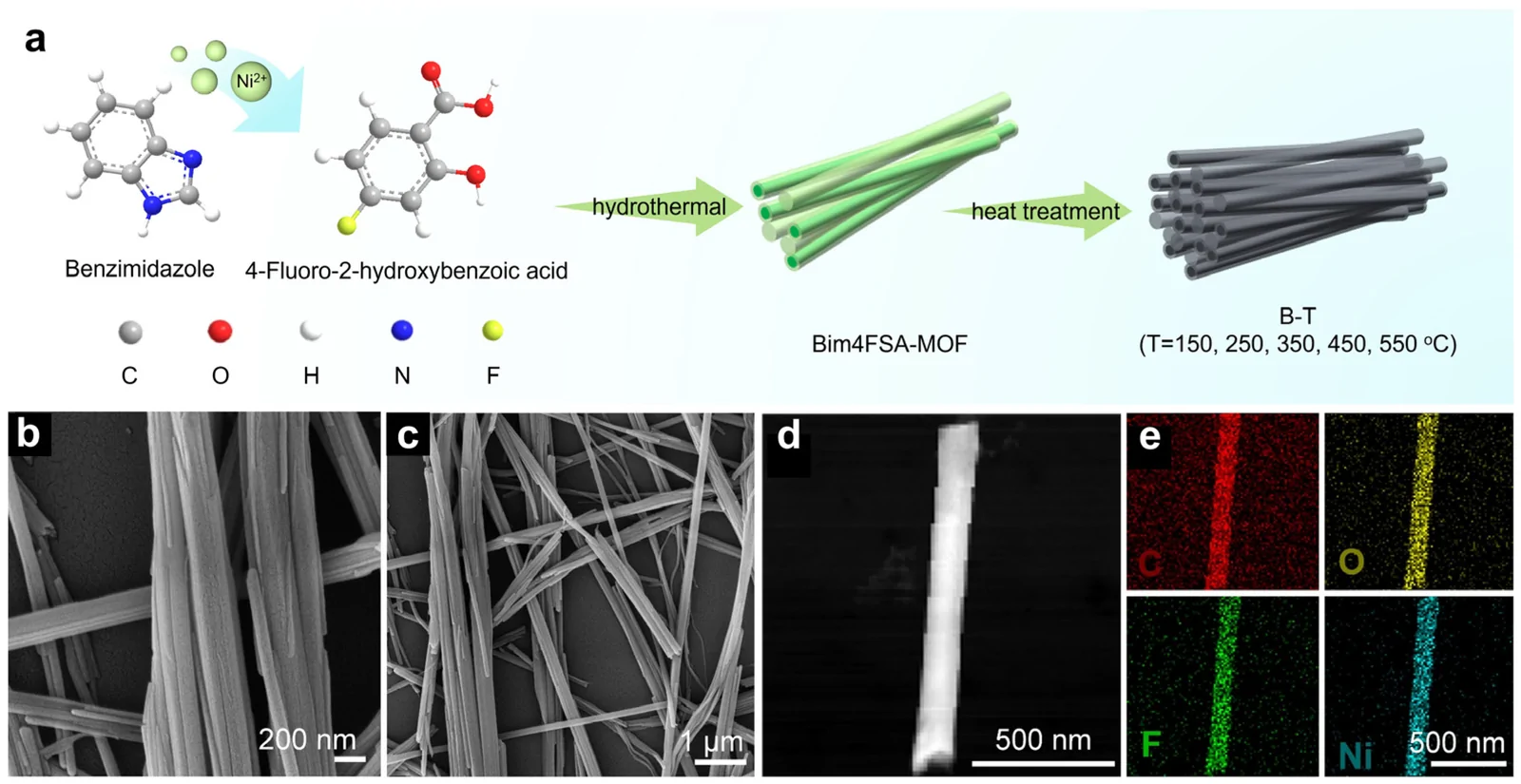
(**a**) Schematic diagram of Bim4FSA-MOF and B-T synthesis. The (**b**,**c**) SEM images, (**d**) HAADF-STEM and (**e**) corresponding element distribution maps of 4FSA-MOF.

As shown in Figure 1a, Bim4FSA-MOF precursor nanorods with uniform element distribution were synthesized by the hydrothermal method using Bim and 4FSA as raw materials in different proportions (Table 1). Subsequently, it was subjected to heat treatment at different temperatures (150–550 °C) to regulate its morphology evolution and form a porous structure (Table 2).

The scanning electron microscope (SEM) images in Figure 1b,c reveal that the 4FSA-MOF is composed of nanorods, forming a one-dimensional (1D) material. This nanorod architecture facilitates tight coordination between the metal ions and organic ligands, endowing the material with enhanced chemical stability and mechanical strength, which is beneficial for maintaining structural integrity during electrochemical measurements [23,24]. Furthermore, the elemental maps in Figure 1d,e demonstrate a uniform distribution of C, O, and F elements derived from the 4FSA ligand, along with Ni originating from the Ni(NO_3_)_2_·6H_2_O precursor [25].

A series of optical images revealed that adjusting the molar ratio of Bim to 4FSA resulted in distinct morphologies of Bim4FSA-MOF. When the initial ratio was set at Q = 1:0.625:0.625, a trace amount of gel-like substance was observed in the reaction mixture (Figure S1a). Upon doubling the amount of Bim (Q = 1:1.25:0.625), the quantity of the gel-like substance increased (Figure S1b). In contrast, when the amount of 4FSA was doubled (Q = 1:0.625:1.25), a large amount of greyish-blue precipitate was formed (Figure S1c). Furthermore, increasing the amounts of both Bim and 4FSA to varying extents led to the formation of flocculent precipitates with different degrees of aggregation (Figure S1d–f). At a ratio of Q = 1:2.5:2.5, a more pronounced change was observed, where the reaction solution became transparent with no apparent product formation (Figure S1g).

**Figure 2:**
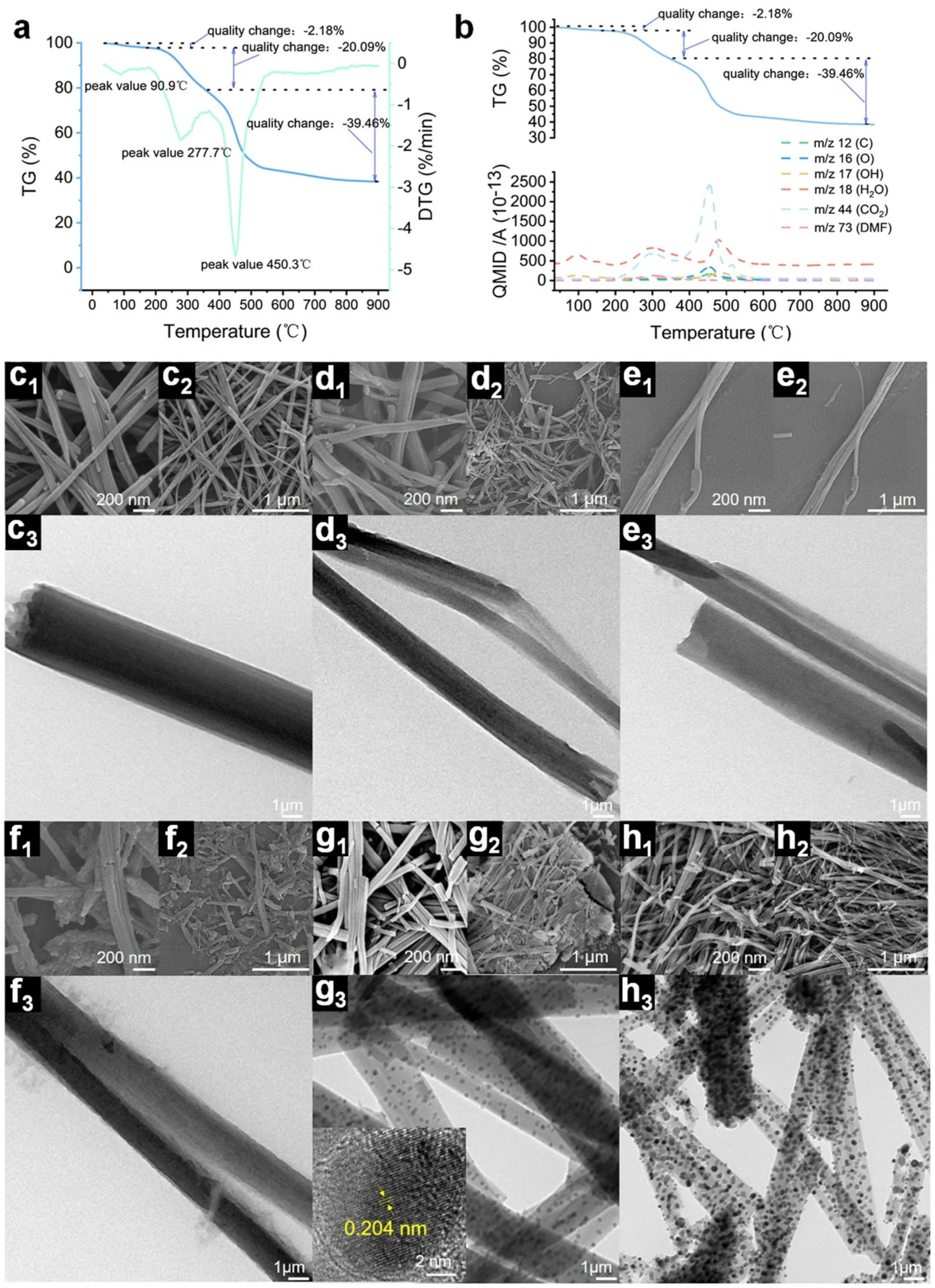
(**a**) TG-DTG and (**b**) TG-MS diagrams of Bim4FSA-MOF-3 sample during heat treatment under nitrogen atmosphere. (**c_1_**,**c_2_**) SEM and TEM (**c_3_**) of Bim4FSA-MOF-3. (**d_1_**,**d_2_**) SEM and TEM (**d_3_**) of B-150. (**e_1_**,**e_2_**) SEM and TEM (**e_3_**) of B-250. (**f_1_**,**f_2_**) SEM and TEM (**f_3_**) of B-350. (**g_1_**,**g_2_**) SEM and TEM (**g_3_**) of B-450 (Illustration is its crystal plane spacing). (**h_1_**,**h_2_**) SEM and TEM (**h_3_**) of B-550.

The microstructures of the Bim4FSA-MOF samples were further characterized by SEM analysis. As shown in Figure S2a,b, Bim4FSA-MOF-1 and Bim4FSA-MOF-2 did not exhibit the nanorod-like morphology typical of 4FSA-MOF, while predominantly forming bulk aggregates. Instead, Bim4FSA-MOF-3 displayed a uniform nanorod-like morphology (Figure 2(c_1_)). Although Bim4FSA-MOF-4−6 exhibited nanorod-like structures with varying morphologies, their formation was accompanied by the presence of agglomerated particles (Figure S2c–e). These results collectively demonstrate that the molar ratio of Bim to 4FSA significantly influences the morphology of the resulting Bim4FSA-MOF [26,27]. To determine an appropriate heat treatment temperature, TG-DTG analysis of Bim4FSA-MOF-3 was performed under N_2_ atmosphere (Figure 2a). As shown by TG-MS data in Figure 2b, decomposition was most rapid around 500 °C. Further heating intensified carbonization of the 4FSA ligand, generating substantial CO_2_ (*m*/*z* = 44) and H_2_O (*m*/*z* = 18), resulting in a final weight loss of 39.46%. Based on the TG-MS and TG-DTG results, thermal treatment was performed at 150, 250, 350, 450, and 550 °C, yielding the B-T products. Optical images (Figure S3) reveal distinct color changes on the material surface after treatment at different temperatures [28,29]. To further investigate microstructural evolution, SEM and transmission electron microscopy (TEM) characterization were conducted.

As shown in Figure 2(c_1_–c_3_), the pristine Bim4FSA-MOF-3 exhibits a nanorod-like morphology similar to that of 4FSA-MOF. After treatment at 150 °C, the nanorods begin to shrink and fragment into short, broken nanorods (Figure 2(d_1_–d_3_)). At 250 °C, the nanorods appear contracted and aggregated (Figure 2(e_1_–e_3_)). Upon increasing the temperature to 350 °C, the nanorod structure transitions to a molten state with partial agglomeration into small clusters (Figure 2(f_1_–f_3_)), indicating significant structural disruption consistent with the TG-MS and TG-DTG results [30]. At higher temperatures (450 °C and 550 °C), more particulates form on the nanorod surfaces, with density increasing at higher temperatures (Figure 2(g_1_–g_3_,h_1_–h_3_)). High-resolution TEM (HRTEM) analysis of B-450 reveals lattice fringes with a spacing of 0.204 nm, corresponding to the (100) plane of metallic Ni (inset of Figure 2(g_3_)), suggesting that these particulates are Ni nanoparticles, which are unfavorable for redox reactions [31]. In summary, thermal treatment at 250 °C optimally preserves the original Bim4FSA-MOF-3 structure while releasing guest molecules from the channels, thereby enlarging pores and facilitating electron transfer to enhance performance [32,33].

**Figure 3:**
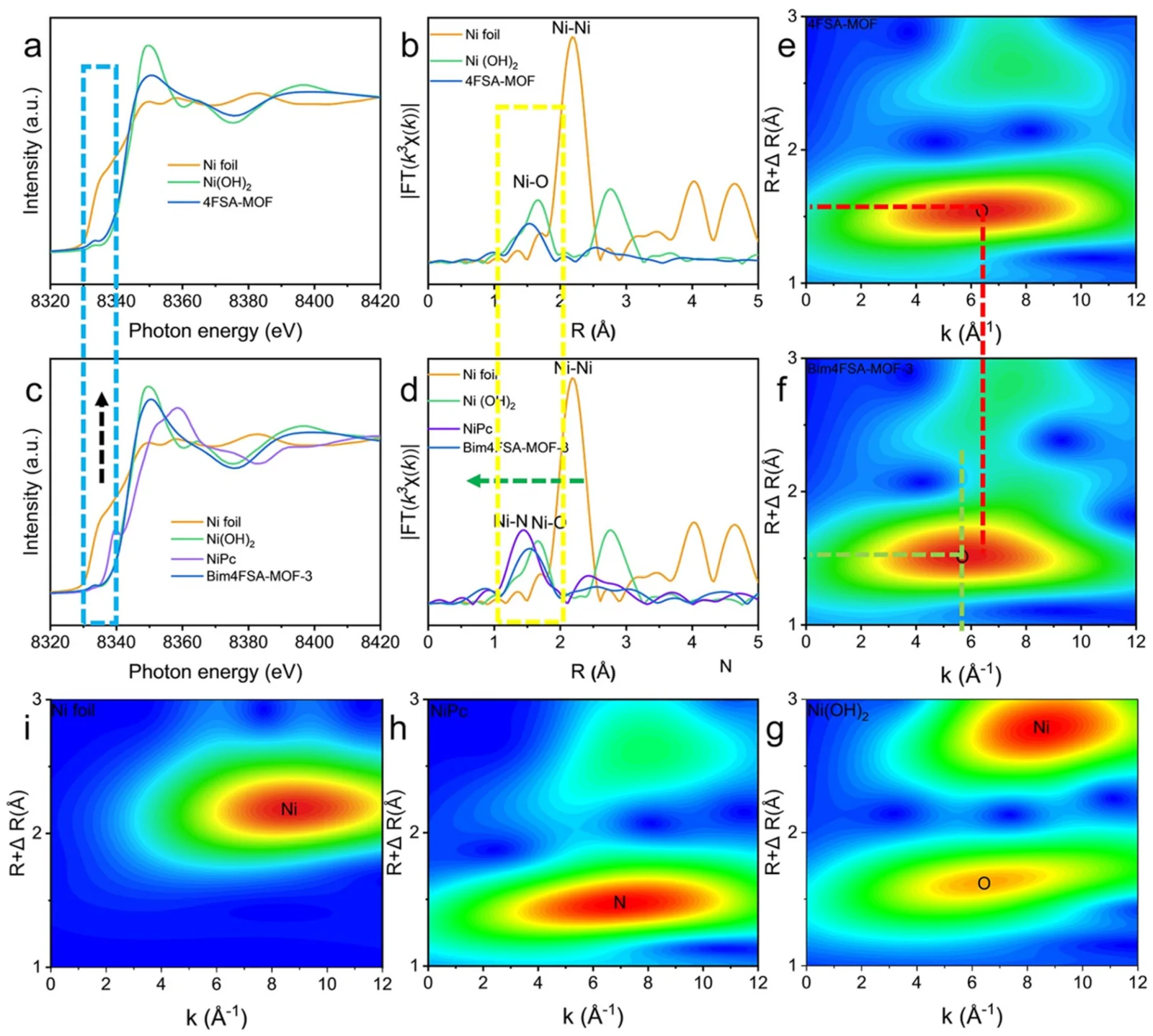
(**a**,**c**) XANES and (**b**,**d**) FT-EXAFS spectra of Ni foil, Ni(OH)_2_, 4FSA-MOF, NiPc and Bim4FSA-MOF-3. WT contour plots of the Ni K-edge for (**e**) 4FSA-MOF, (**f**) Bim4FSA-MOF-3, (**g**) Ni(OH)_2_, (**h**) NiPc and (**i**) Ni foil, respectively.

The Ni-k edge XANES spectra in Figure 3a,c show the local structure information of 4FSA-MOF and Bim4FSA-MOF-3. Compared with Ni foil and Ni(OH)_2_, their Ni-k edge absorption energy position is higher than that of Ni foil, and the center is closer to Ni(OH)_2_ (Ni^2+^), indicating that the Ni atom is positively charged [34]. Comparing the front edge positions of 4FSA-MOF and Bim4FSA-MOF-3, a significant peak enhancement at about 8332 eV corresponds to the dipole-forbidden transition from 1s to 3d, which further indicates that the Ni coordination environment is not completely centrosymmetric. The strength of Bim4FSA-MOF-3 in the dipole-forbidden region is significantly weaker than that of 4FSA-MOF (in the blue box), indicating that the Bim in Bim4FSA-MOF-3 has an effect on the Ni−O coordination mode of its original 4FSA-MOF, and the nitrogen in Bim is involved in the coordination. Figure 3b shows that 4FSA-MOF has a significant peak near 1.55 Å, which is close to the Ni−O distance in Ni(OH)_2_, further confirming that the coordination environment of Bim4FSA-MOF is mainly composed of Ni−O bonds.

Nevertheless, the Ni–O bond in Bim4FSA-MOF-3 shifts toward the low-spacing direction of the Ni–N bond (Figure 3d, yellow box), and the coordination mode at this stage is more inclined toward Ni–O. The trace Ni–N bond exerts a regulatory influence on the Ni–O bond. Finally, wavelet-transform analysis (Figure 3e–i) shows that the Ni–O bond in 4FSA-MOF (red traces) undergoes a more pronounced shift than that in Bim4FSA-MOF-3 (green traces), reflecting the influence of Bim on bond regulation and suggesting a trend toward shorter bond lengths and enhanced structural stability in Bim4FSA-MOF-3.

The structure and properties of Bim4FSA-MOF-3 samples after heat treatment were analyzed by XRD, Raman, Zeta potential and pore size distribution. As shown in Figure S4a, the as-prepared Bim4FSA-MOF-3 sample exhibits obvious XRD diffraction peaks at 2θ < 10° determined by its large unit cell parameters and pore structure, which is usually found in MOF/coordination polymer materials. The XRD patterns of B-150 and -250 are consistent with those of Bim4FSA-MOF-3 after heat treatment before 250 °C, indicating that heat treatment temperatures less than or equal to 250 °C will not lead to structural changes. However, when the temperature rises to 350 °C, the intensity of some characteristic peaks is weakened. After 450 °C, the main characteristic peaks of Bim4FSA-MOF-3 disappeared, accompanied by the characteristic peaks of elemental nickel (PDF#87-0712). Raman spectroscopy in Figure S4b shows that the structure is stable after heat treatment below 250 °C. At 350 °C, the Raman spectrum of the Bim4FSA-MOF-3 sample has a large noise signal, indicating that the material skeleton is damaged [35]. When the temperature exceeds 450 °C, the intensity of the D band (light blue) and the G band (light pink) in the Raman spectrum is significantly enhanced, indicating that the carbon-based material forms a small amount of half-ion C−F bonds, which partially compensates for the performance degradation caused by Ni formation. In Figure S4c, as the temperature increases, the Zeta potential becomes more negative (measured in aqueous dispersion), suggesting a more negative surface charge that indicates improved colloidal dispersion stability in this temperature range. When the temperature exceeds 450 °C, the Zeta potential begins to move to a positive value, and the surface charge electricality is reversed.

To further reveal the change in pore size after heat treatment, the data were summarized in Table S1. It was found that the pore size increased from 12.03 to 20.13 nm when the heat treatment temperature rose from room temperature to 250 °C. When the temperature exceeds 250 °C, the sample still maintains a one-dimensional rod-shaped/nanowire main morphology; the pore size shrinks slightly as elemental nickel forms on the surface and blocks the pores. This demonstrates that removing guest molecules from the pores via heat treatment is a feasible strategy for tuning pore size, which can expand the pore channels, promote the release of the active sites, accelerate the electron transfer, and thereby further enhance the electrochemical performance [36,37].

### 2.2. Electrochemical Performance

**Figure 4:**
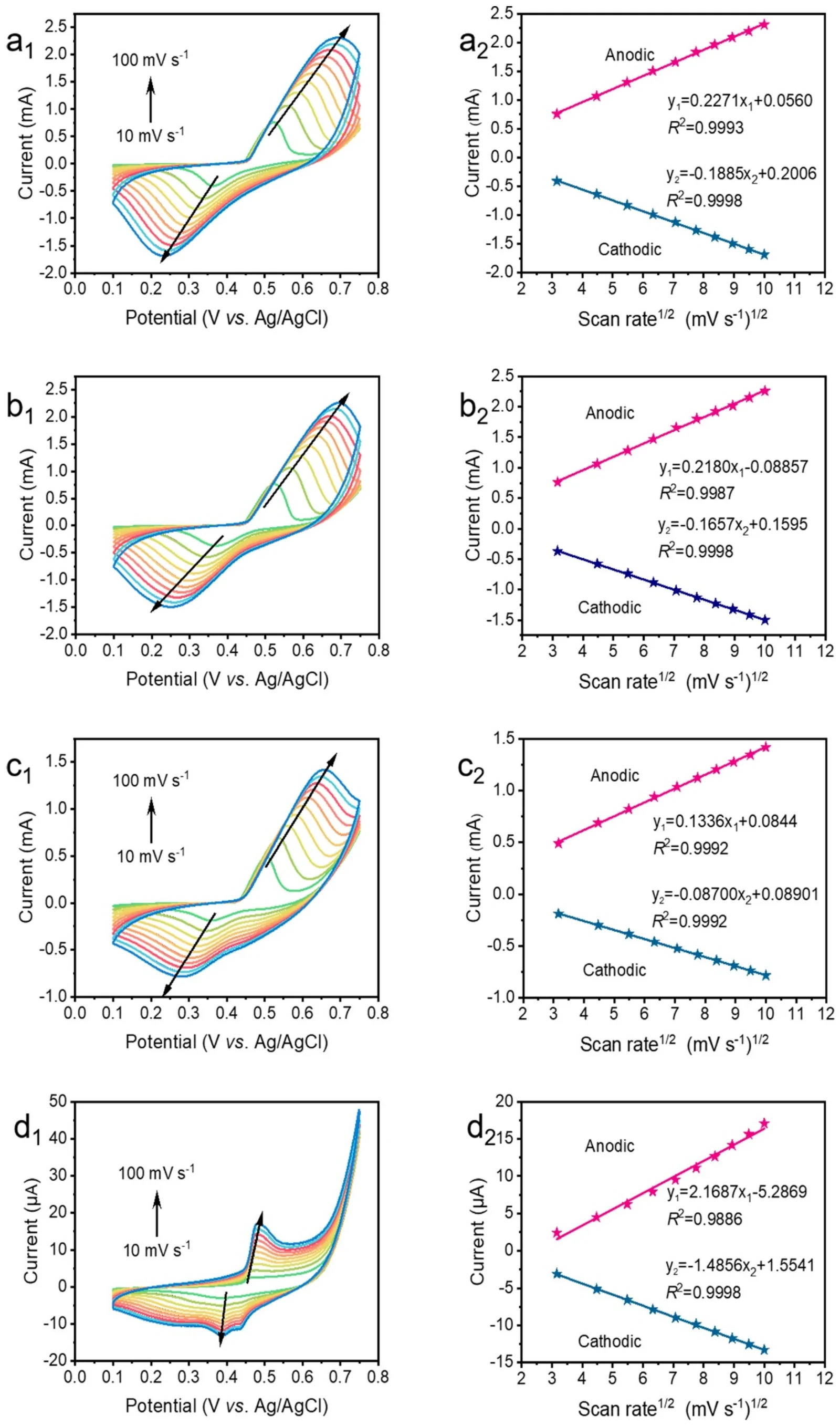
CV curves of (**a_1_**,**a_2_**) B-150, (**b_1_**,**b_2_**) B-250, (**c_1_**,**c_2_**) B-450 and (**d_1_**,**d_2_**) B-550 in 0.1 M NaOH solution at scan rates from 10 to 100 mV s^−1^, along with the linear relationships between the oxidation/reduction peak currents and scan rate^1/2^.

The glucose sensor was measured in 0.1 M NaOH solution with 4FSA-MOF and Bim4FSA-MOF-3 as the active electrode materials, and their CV curves were measured at various scan rates, as shown in Figure S5(a_1_,b_1_). As the scan rate increased, the internal diffusion resistance of the samples also increased, leading to a corresponding increase in current. Meanwhile, the oxidation and reduction peaks shifted to more positive and more negative potentials, respectively. As observed in Figure S5(a_2_,b_2_), the R^2^ values between the peak current density and the square root of the scan rate were all above 0.99, indicating a diffusion-controlled electrochemical process. In Figure 4(a_1_–d_1_), the overall variation process was similar to that of Bim4FSA-MOF-3, suggesting a diffusion-controlled process, although the thermal treatment significantly altered the response current intensity and the corresponding linear relationship [38]. In the linear relationships shown in Figure 4(a_2_–d_2_), it was evident that the R^2^ values of the thermally treated samples improved to varying degrees. The current intensities of B-150 and B-250 were significantly higher than those of B-450 and B-550. This phenomenon is ascribed to the thermal treatment of B-150 and B-250, which opened up the pores and facilitated the release of active sites. In contrast, the structures of B-450 and B-550 were damaged, accompanied by the formation of elemental nickel, leading to decreased performance. Higher temperatures resulted in more elemental nickel and a more rapid decline in performance.

Figure S6a–f illustrates the electrochemical oxidation processes of glucose on different electrode materials. While the current variation (measured in mA) induced by the addition of varying concentrations of glucose was not particularly pronounced, a notable increase in peak current for B-250 was clearly observed in the inset of Figure S6d. At a scan rate of 50 mV·s^−1^, the oxidation peak for B-250 was positioned around 0.60–0.66 V. In contrast, the oxidation peaks for B-450 and B-550 shifted toward lower potentials, and their current intensities were significantly weaker compared to those of B-150 and B-250. This indicates that excessive pyrolysis temperatures adversely affect the final electrochemical performance. Optimal working potentials vary among different materials. To determine these potentials, I–T curves were recorded by successively adding 10 µL of 0.1 M glucose at 50 s intervals. Based on the results shown in Figure S7, the optimal potentials for 4FSA-MOF, Bim4FSA-MOF-3, B-150, B-250, B-450, and B-550 were identified as 0.65 V, 0.65 V, 0.66 V, 0.66 V, 0.64 V, and 0.49 V, respectively.

**Figure 5:**
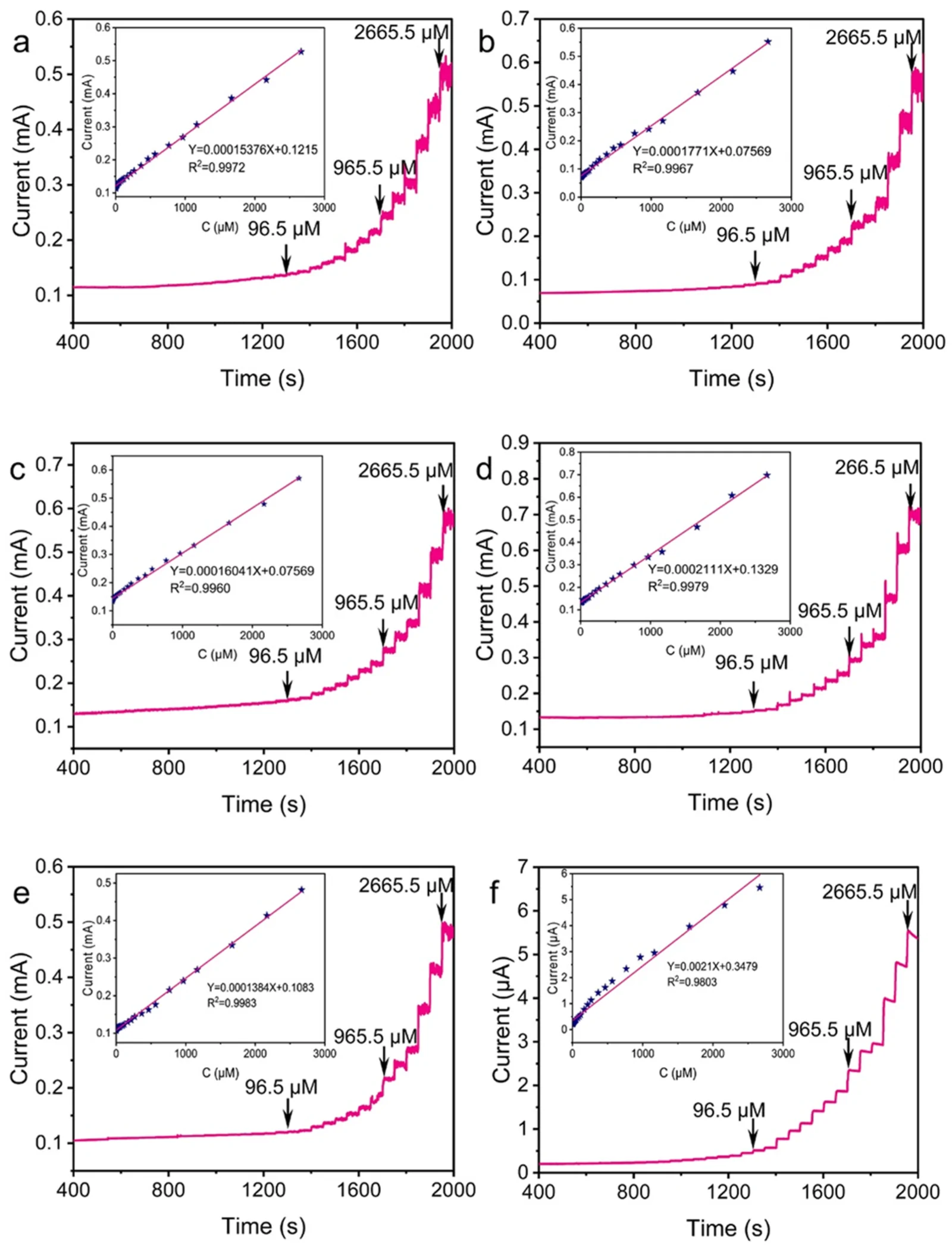
I-T curves of (**a**) 4FSA-MOF, (**b**) Bim4FSA-MOF-3, (**c**) B-150, (**d**) B-250, (**e**) B-450 and (**f**) B-550 at the optimal voltage in 0.1 M NaOH with different concentrations of Glu.

The I-T plots in Figure 5a–f show a distinct step-like current response for glucose in the range of 0.5–2665.5 μM. The current response value of B-250 is the highest. The insets in Figure 5a–f are linear fitting equations. B-250 obtained at 250 °C has the highest sensitivity of 2986.45 μA mM^−1^ cm^−2^ and the lowest detection limit of 0.022 μM. Both the sensitivity and detection limit of B-250 are improved compared to other electrode materials (Table S2). This improvement in performance is attributed to the fact that the heat treatment process at 250 °C maximally preserves the morphological characteristics of the original MOF [39]. The heat treatment is proposed to activate the nickel-based metal sites in B-250 and remove the guest molecules in the pores, expanding the pores, which is beneficial for the entry of glucose molecules and the improvement of the electron transfer rate [40,41]. When the temperature is 450 and 550 °C, the performance declines rapidly due to the large-scale production of elemental nickel on the surface.

### 2.3. Mechanism Analysis

**Figure 6:**
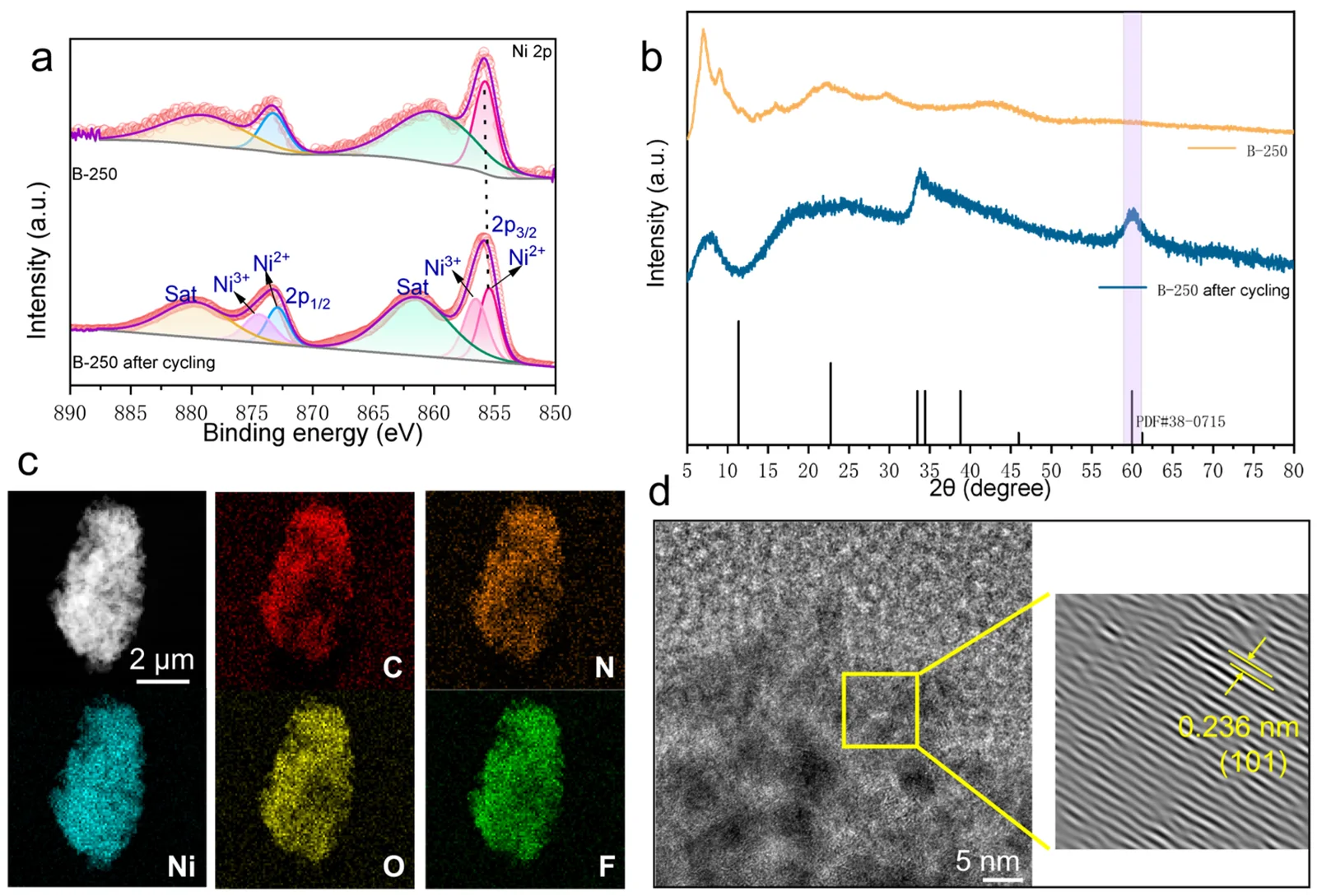
(**a**) B-250 test before and after the XPS; (**b**) XRD patterns of B-250 before and after testing; (**c**) Elemental mapping images of B-250 after testing; (**d**) HRTEM image of B-250 after testing and the corresponding magnified view.

As shown in Figure 6a, the peaks at 856.57 eV and 874.42 eV in the Ni 2p spectrum represent Ni^3+^, which is formed due to the partial conversion of Ni(OH)_2_ to NiOOH during the electrochemical cycle (Equations (1)–(6)). This process provides more activity for the electrochemical oxidation of glucose and is a dynamic process of active site transformation. To further investigate the glucose oxidation process on the B-250 modified electrode material, XRD analysis in Figure 6b provides additional insights into the structural changes in B-250 following long-term cycling tests. After testing, the diffraction peaks of B-250 were attenuated, and a new diffraction peak emerged at 59.98°, corresponding to the formation of Ni(OH)_2_·0.75H_2_O (PDF#38-0715). This indicates a compositional modification of the material during the electrochemical cycling process [42]. The chemical processes occurring on the electrode are represented by Equation (1) to Equation (6), where the generation of Ni(OH)_2_ originates from OH^−^ (derived from H_2_O and NaOH in the electrolyte) and Ni^2+^ present in B-250.It should be noted that, since no distinct characteristic peaks of metallic nickel were observed in the XRD pattern after the test, Equation (5) is retained only as a tentative pathway.

NaOH → Na^+^+ OH^−^ (1)

H_2_O ⇌ H^+^ + OH^−^ (2)

2H^+^ + 2e^−^ = H_2_ (3)

Ni^2+^ + 2OH^−^ → Ni(OH)_2_ (4)

Ni^2+^ + 2e^−^ = Ni (5)

Ni(OH)_2_ + OH^−^ → NiOOH + H_2_O + e^−^ (6)

As shown in Figure 6d, prominent lattice fringes with an interplanar spacing of 0.236 nm are observed, which corresponds precisely to the (101) crystallographic plane of Ni(OH)_2_. These structural observations corroborate the mechanistic interpretation derived from the electrochemical equations presented earlier, supporting the conclusion that B-250 undergoes conversion to Ni(OH)_2_ during electrochemical testing (these images were acquired after the electrochemical test and thus reflect the post-test state). Moreover, elemental mapping (Figure 6c) reveals uniform spatial distribution of all constituent elements, with no obvious segregation or large-scale element loss observed in the elemental maps, further hinting at the potential stabilizing role of fluorinated organic ligands and controlled thermal treatment in preserving the structural and compositional integrity of B-250 (Figure S8). According to the nitrogen adsorption–desorption isotherm and pore size distribution diagram in Figure S9, with the increase in heating temperature, the isotherm shape and hysteresis loop of the Ni-MOF sample change significantly, indicating that the pore structure gradually evolves.

When 100 μM glucose was added to 0.1 M NaOH, the B-250 material showed a significant current response (Figure S10). Then, the interfering factors DA, UA, AA and NaCl were introduced in sequence, and each interfering substance was maintained throughout the system. The concentrations of both were 5 μM. After the introduction of interfering substances, there was no obvious change in the electrochemical response of the material. Add 100 μM glucose again, the corresponding electrochemical response was restored, indicating that B-250 is effective against common interfering species with excellent anti-interference performance.

## 3. Materials and Methods

### 3.1. Chemicals

Nickel (II) nitrate hexahydrate (Ni(NO_3_)_2_·6H_2_O), *N*,*N*-dimethylformamide (DMF), ethanol were purchased from Shanghai Sinopharm Chemical Reagent (Shanghai, China). Benzimidazole (Bim, 99.5%), 4-fluorosalicylic acid (4FSA), glucose (Glu, 99.5%), sodium hydroxide (NaOH, 97%) was purchased from Aladdin (Shanghai, China). All chemicals were of analytical grade and used without further purification. Deionized water was used in all experiments.

### 3.2. Synthesis of 4FSA-MOF

Ni(NO_3_)_2_·6H_2_O (0.5901 g) and 4FSA (0.5940 g) were weighed and dissolved in 60 mL of DMF under thorough stirring to obtain solution A. Subsequently, 6 mL of 0.4 M NaOH solution was added dropwise to solution A over a period of 10 min. After the addition, the mixture was stirred for an additional 30 min to obtain solution B. The resulting solution B was transferred into a 100 mL Teflon-lined stainless steel autoclave and maintained at 100 °C for 8 h. After the reaction, the product was washed three times with DMF and ethanol, respectively, and then dried at 80 °C for 12 h to obtain 4FSA-MOF.

### 3.3. Synthesis of Bim4FSA-MOF

Ni(NO_3_)_2_·6H_2_O (0.5901 g), Bim (0.1499 g), and 4FSA (0.3959 g) were weighed and dissolved in 60 mL of DMF under thorough stirring to obtain solution C. Subsequently, 6 mL of 0.4 M NaOH solution was added dropwise to solution C over a period of 10 min. After the addition, the mixture was stirred for an additional 30 min to obtain solution D. The resulting solution D was transferred into a 100 mL Teflon-lined stainless steel autoclave and maintained at 100 °C for 8 h. After the reaction, the product was washed three times with DMF and ethanol, respectively, and then dried at 80 °C for 12 h to obtain Bim4FSA-MOF-3.

Bim4FSA-MOF samples with different Bim/4FSA ratios were synthesized following a procedure similar to that of Bim4FSA-MOF, except that the amounts of Bim and 4FSA added were varied, as specified in Table 1.

### 3.4. Synthesis of B-T

100 mg (0.308 mmol) of Bim4FSA-MOF-3 sample was dispersed in a porcelain boat, sealed in a quartz tube, and the air in the tube was replaced with a nitrogen atmosphere for 30 min. The exhaust gas was connected to the water, and the N_2_ flow rate was controlled at about 20 mL min^−1^. After full replacement, the heat treatment was started at a heating rate of 1 °C min^−1^. When the temperature was raised to T °C, the heating was stopped (no dwell/holding at T °C was applied), the sample was allowed to cool naturally, and the nitrogen atmosphere was maintained during the cooling process. T = 150, 250, 350, 450 and 550; the specific details are shown in Table 2, and the post-processing method remains unchanged.

## 4. Conclusions

A series of Bim4FSA-MOF composites were synthesized using a solvothermal method and subsequent controlled thermal treatment. The composite thermally treated at 250 °C (B-250) retained a well-defined one-dimensional nanowire morphology (though with slight contraction and aggregation relative to the precursor), which is proposed to facilitate short electron-transfer pathways and an increased accessible surface area. This structure is expected to benefit conductivity and the exposure of active nickel sites. Consequently, electrodes fabricated with B-250 exhibited notable non-enzymatic glucose-sensing performance, characterized by a low detection limit of 0.022 μM, a high sensitivity of 2986.45 μA mM^−1^ cm^−2^, and a rapid response time of 0.9 s. These superior performances are attributed to the unique nanowire architecture in conjunction with the in situ generated Ni(OH)_2_/NiOOH dynamic active centers. This research provides significant insights into the rational design of high-performance one-dimensional MOF electrocatalysts inferred from morphology and multiple characterizations through the integration of morphological control, coordination modulation, and post-synthetic activation for advanced sensing interfaces.

## Figures and Tables

**Table 1:** The specific addition amount of Bim and 4FSA in different proportions.

Samples	Ni(NO_3_)_2_·6H_2_O (g/mmol)	Bim (g/mmol)	4FSA (g/mmol)	Molar Ratio
Bim4FSA-MOF-1	0.5901/2.0290	0.1499/1.2690	0.1980/1.2680	1:0.625:0.625
Bim4FSA-MOF-2	0.5901/2.0290	0.2998/2.5380	0.1980/1.2680	1:1.25:0.625
Bim4FSA-MOF-3	0.5901/2.0290	0.1499/1.2690	0.3960/2.5370	1:0.625:1.25
Bim4FSA-MOF-4	0.5901/2.0290	0.2998/2.5380	0.3960/2.5370	1:1.25:1.25
Bim4FSA-MOF-5	0.5901/2.0290	0.5996/5.0750	0.3960/2.5370	1:2.5:1.25
Bim4FSA-MOF-6	0.5901/2.0290	0.2998/2.5380	0.7920/5.0730	1:1.25:2.5
Bim4FSA-MOF-7	0.5901/2.0290	0.5996/5.0750	0.7920/5.0730	1:2.5:2.5

**Table 2:** Abbreviations and Reaction Conditions of samples.

Samples	Bim4FSA-MOF-3 (mg/mmol)	T (°C)
B-150	100/0.308	150
B-250	100/0.308	250
B-350	100/0.308	350
B-450	100/0.308	450
B-550	100/0.308	550

## Data Availability

The data that support the findings of this study are available from the corresponding authors upon reasonable request.

## Supplementary Materials

The following supporting information can be downloaded at https://www.mdpi.com/article/10.3390/molecules31173096/s1, Figure S1: Different ratios of Bim4FSA-MOF are presented from left to right: (a) Bim4FSA-MOF-1, (b) Bim4FSA-MOF-2, (c) Bim4FSA-MOF-3, (d) Bim4FSA-MOF-4, (e) Bim4FSA-MOF-5, (f) Bim4FSA-MOF-6, and (g) Bim4FSA-MOF-7; Figure S2: SEM images of (a) Bim4FSA-MOF-1, (b) Bim4FSA-MOF-2, (c) Bim4FSA-MOF-4, (d) Bim4FSA-MOF-5 and (e)Bim4FSA-MOF-6; Figure S3: Optical photographs of B-T after heat treatment at different temperatures; Figure S4: (a) XRD patterns, (b) Raman spectra, and (c) Zeta potential plots of Bim4FSA-3, B-150, B-250, B-350, B-450, and B-550; Figure S5: CV curves of (a_1_,a_2_) 4FSA-MOF and (b_1_,b_2_) Bim4FSA-MOF-3 in 0.1 M NaOH solution at scan rates from 10 to 100 mV s^−1^, along with the corresponding linear relationships between the oxidation/reduction peak currents and scan rate^1/2^; Figure S6: CV curves of (a) 4FSA-MOF, (b) Bim4FSA-MOF-3, (c) B-150, (d) B-250, (e) B-450, and (f) B-550 in 0.1 M NaOH solution at various concentrations; Figure S7: I-T curves of (a) 4FSA-MOF, (b) Bim4FSA-MOF-3, (c) B-150, (d) B-250, (e) B-450, and (f) B-550 recorded in 0.1 M NaOH with successive additions of 10 µL of 0.1 M glucose at 50 s intervals under different applied potentials; Figure S8: (a) XPS survey spectra, (b) high-resolution C 1s, (c) O 1s, and (d) Ni 2p XPS spectra of the Bim4FSA MOF-3 and B-250 samples; Figure S9: (a–f) The nitrogen adsorption-stripping isothermal line (illustrations for pore size distribution curve) of Bim4FSA-MOF-3, Bim4FSA-MOF-150, Bim4FSA-MOF-250, Bim4FSA-MOF-350, Bim4FSA-MOF-450 and Bim4FSA-MOF-550; Figure S10: B-250 interference test curve. Table S1: Pore size distribution of samples heat-treated at different temperatures; Table S2: Comparison of other nickel-based MOFs and non-enzymatic glucose sensors. References [43,44,45,46,47] are cited in the Supplementary Materials.
