# Supplementary material for: Benzimidazole-Regulated 1D 4-Fluorosalicylic Acid MOF Composite Material Design and Its Application in Glucose Sensing
Source: Molecules. 2026 Sep 3;31(17):3096. doi: 10.3390/molecules31173096 (PMC13567323; doi:10.3390/molecules31173096)
Supplement: Supplementary file 1 [file molecules-31-03096-s001.zip › molecules-4463226-supplementary.pdf]

Supporting Information

Benzimidazole-Regulated 1D 4-Fluorosalicylic Acid MOF Composite Material Design and Its Application in Glucose Sensing

Haixia Wu[1], Dianheng Yu[2], Jinliang Hu[2], Fang Wang[2], Songtao Zhang[2,], Kailu Guo[1,*] and Huan Pang[2,3,*]*

[1]College of Chemistry and Chemical Engineering, Henan Key Laboratory of Function-Oriented Porous Materials, Luoyang Normal University, Luoyang, 471934, P. R. China

[2]School of Chemistry and Materials, Testing Center, Yangzhou University, Yangzhou, 225009, P. R. China.

[3]Yangzhou Key Laboratory of Smart Materials and Clean Energy, Interdisciplinary Research Center for Advanced Energy, Yangzhou, 225002, Jiangsu, P. R. China.

E-mail: zhangsongtao@yzu.edu.cn; guokl18@163.com; huanpangchem@hotmail.com

1. Material Characterization

Scanning electron microscope (SEM) and transmission electron microscope (TEM) images were collected on Zeiss Supra 55 and JEM-2100, respectively. The Tecnai G2 F30 transmission electron microscope was used to obtain high-resolution TEM (HRTEM) images, energy-dispersive X-ray spectrum (EDX) mapping, and scanning TEM images with an acceleration voltage of 300 kV. X-ray diffraction (XRD) characterization was carried out on Bruker AXS D8. The chemical states were measured using an X-ray photoelectron spectrometer (XPS, ThermoFisher Scientific ESCALAB 250Xi). Thermogravimetric analysis (TGA) was performed on a PerkinElmer Pyris 1 thermogravimetric analyzer. N_2 adsorption-desorption isotherms were measured at 77 K on a Micromeritics ASAP 2460 surface area and porosimetry analyzer. Additionally, extended X-ray absorption fine structure (EXAFS) measurements were performed at beamline BL14W1 at the Shanghai Synchrotron Radiation Facility.

2. Electrochemical characterizations

In a typical process, 5 mg of active materials, 665 μL of deionized water, 335 μL of C_2H_5OH, and 30 μL of Nafion solution (5 wt%) were mixed and ultrasonicated to obtain catalysis ink. Then, 5 μL of the catalysis ink was dropped on the glassy carbon electrodes with an area of 0.07069 cm^2 and dried naturally at room temperature for 24 h. All the electrochemical characterizations were conducted on a CHI-760E electrochemical workstation by using 0.1 M NaOH as electrolyte, an Ag/AgCl electrode as reference electrode and a Pt foil as counter electrode.

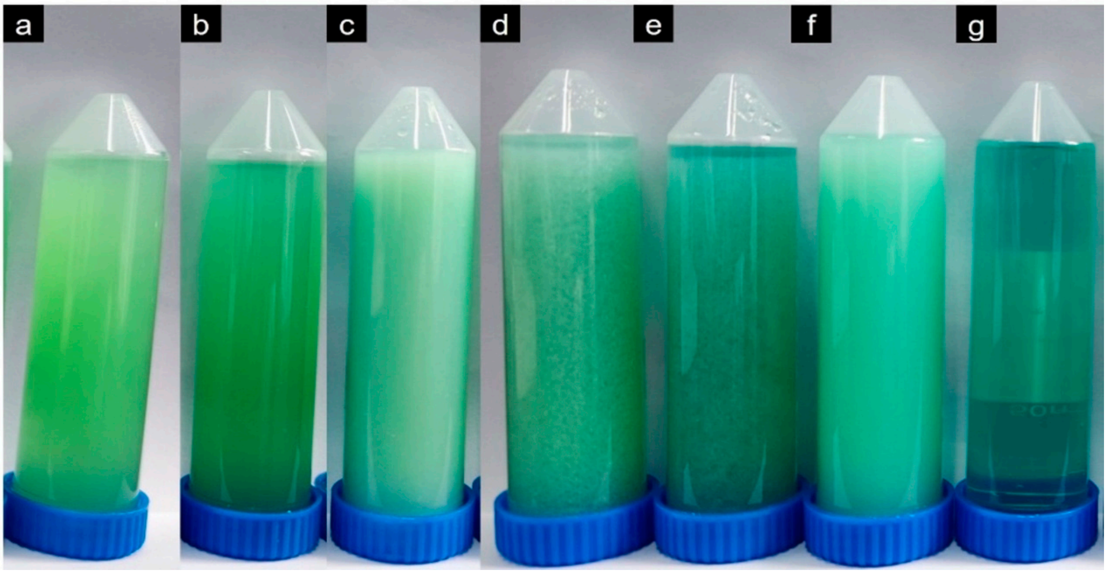

Figure S1. Different ratios of Bim4FSA-MOF are presented from left to right: (a) Bim4FSA-MOF-1, (b) Bim4FSA-MOF-2, (c) Bim4FSA-MOF-3, (d) Bim4FSA-MOF-4, (e) Bim4FSA-MOF-5, (f) Bim4FSA-MOF-6, and (g) Bim4FSA-MOF-7.

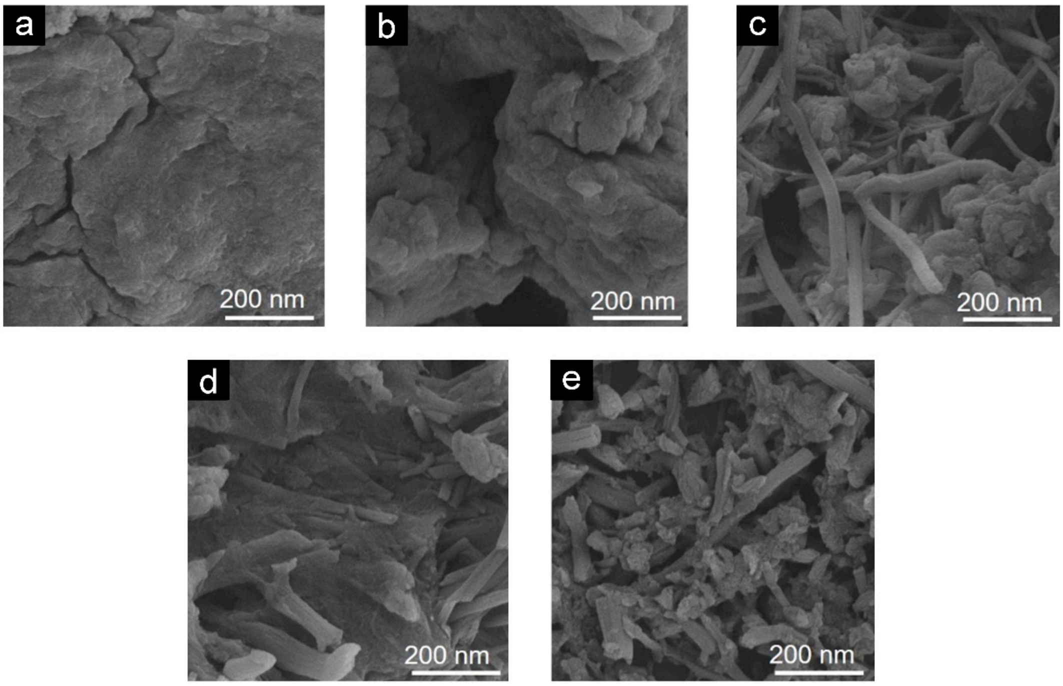

Figure S2. SEM images of (a) Bim4FSA-MOF-1, (b) Bim4FSA-MOF-2, (c) Bim4FSA-MOF-4, (d) Bim4FSA-MOF-5 and (e)Bim4FSA-MOF-6.

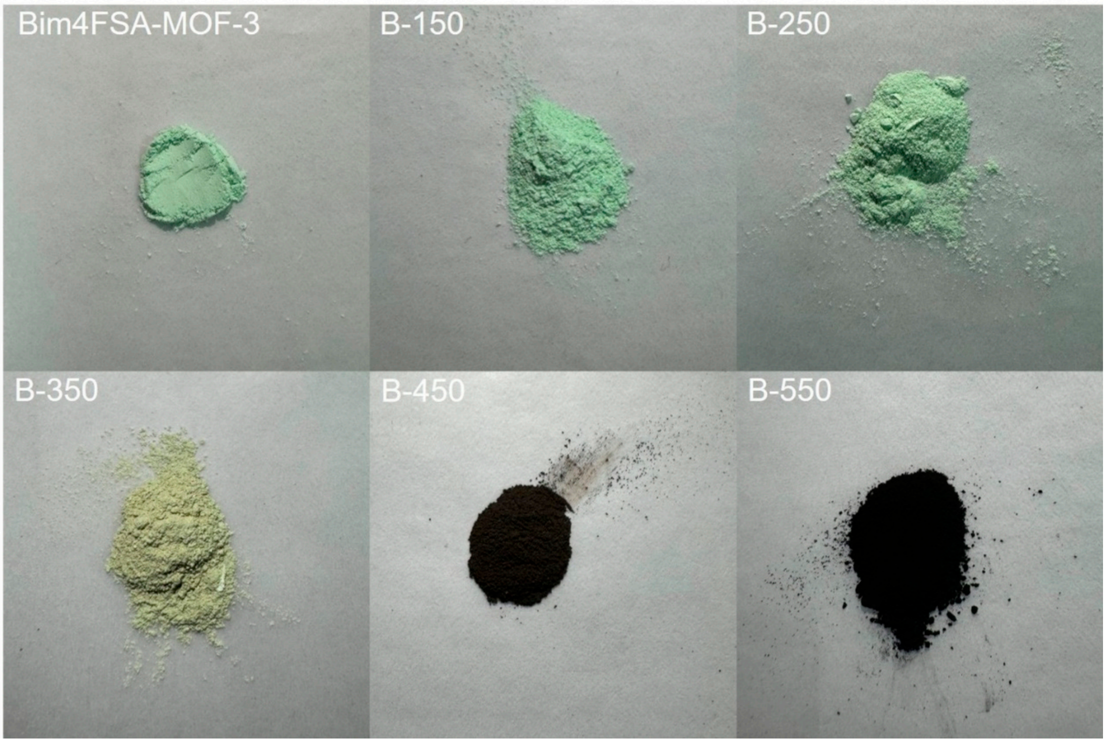

Figure S3. Optical photographs of B-T after heat treatment at different temperatures.

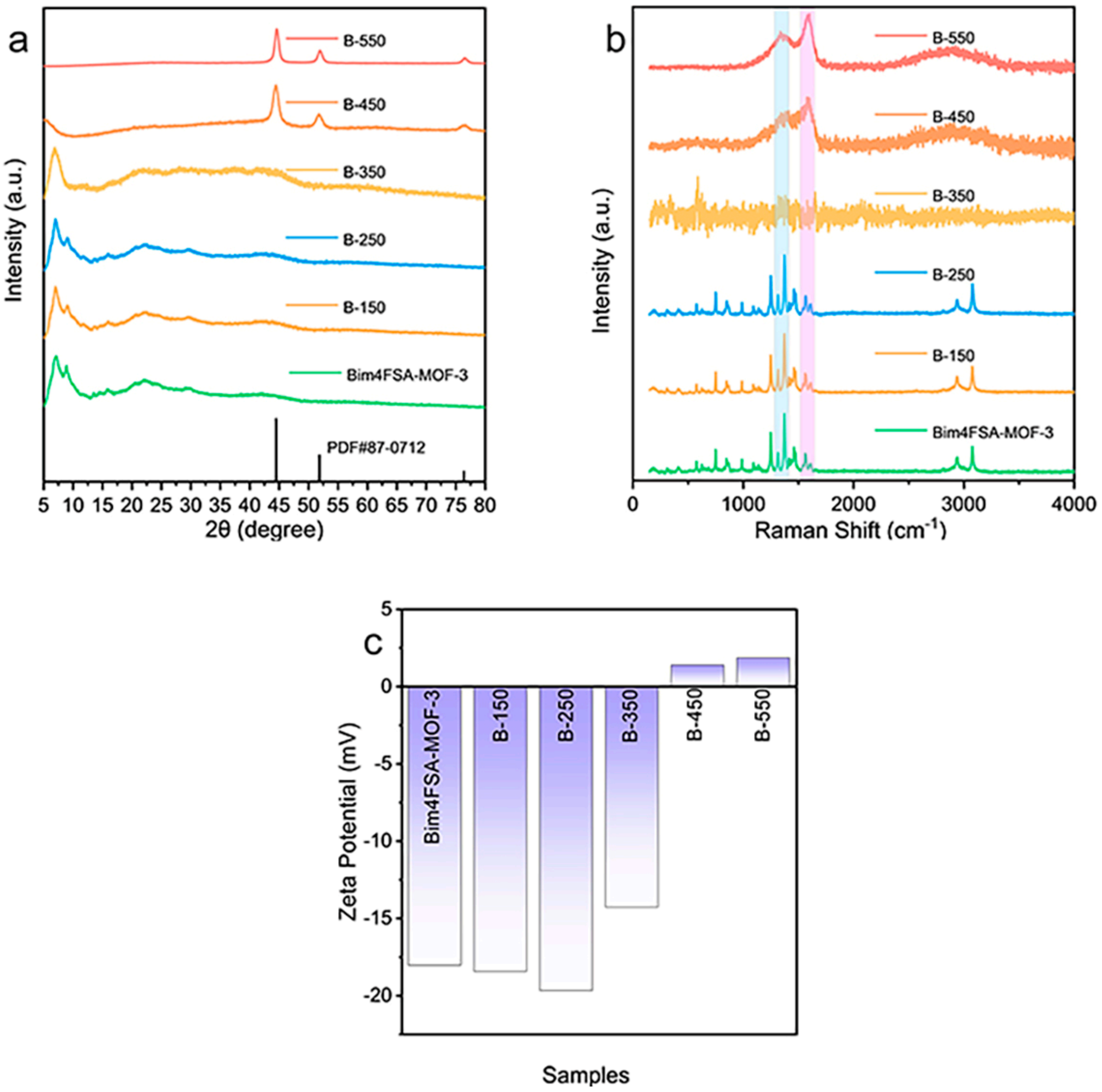

Figure S4. (a) XRD patterns, (b) Raman spectra, and (c) Zeta potential plots of Bim4FSA-3, B-150, B-250, B-350, B-450, and B-550.

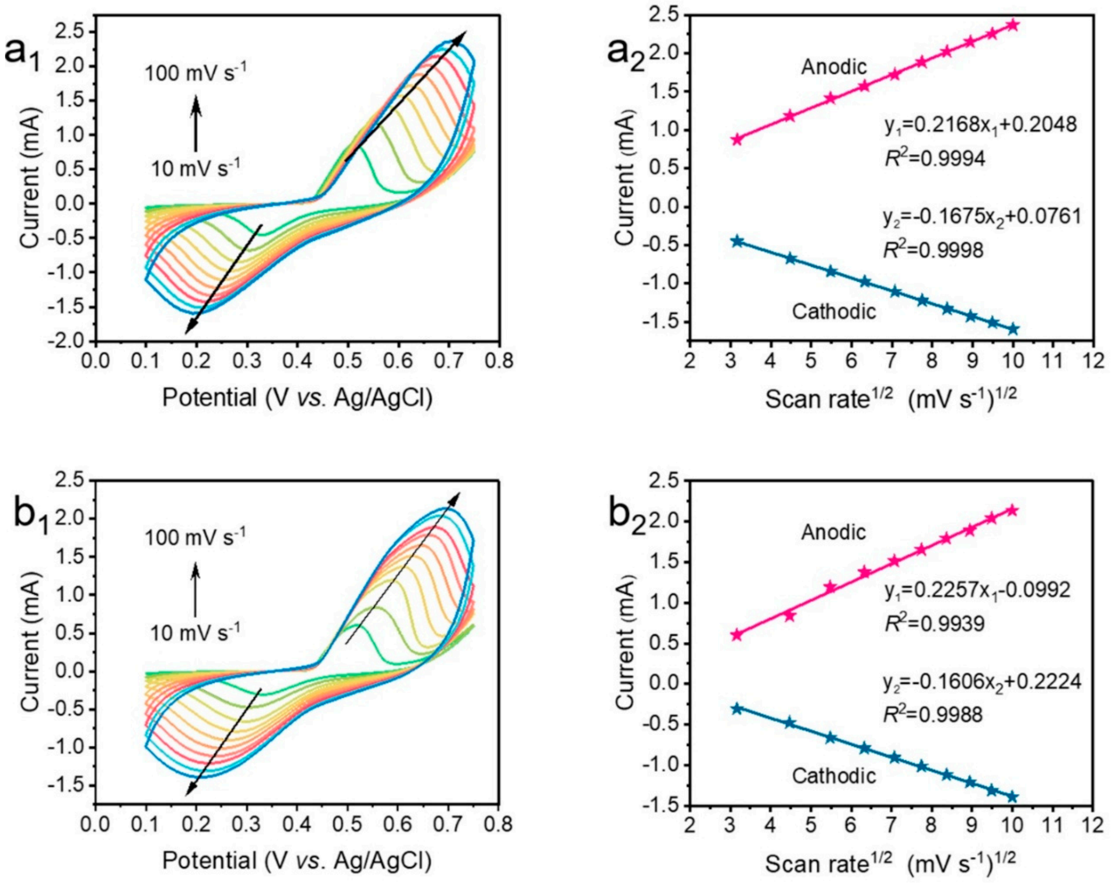

Figure S5. CV curves of (a_1, a_2) 4FSA-MOF and (b_1, b_2) Bim4FSA-MOF-3 in 0.1 M NaOH solution at scan rates from 10 to 100 mV s^{-1}, along with the corresponding linear relationships between the oxidation/reduction peak currents and scan rate$^{1/2}$.

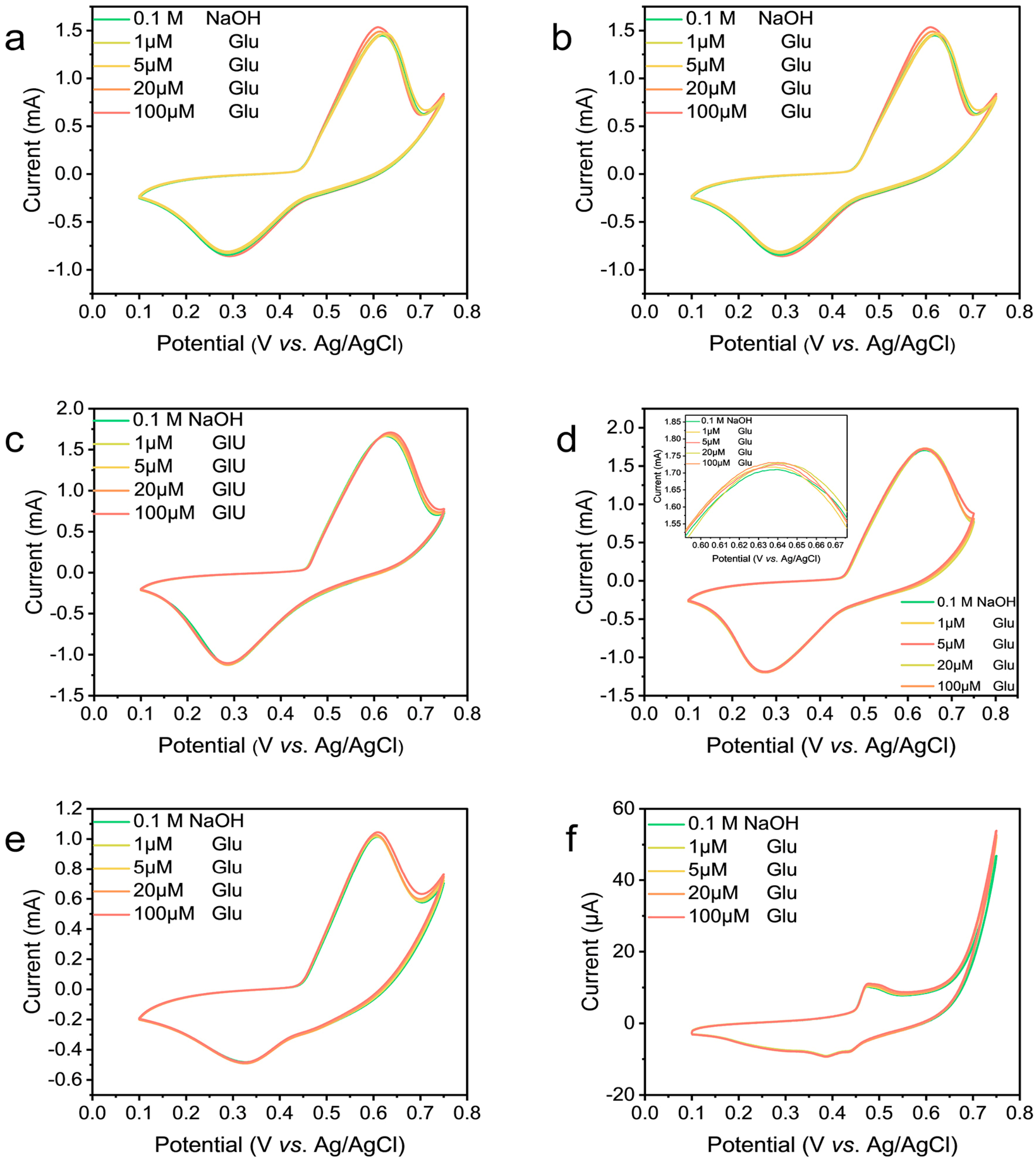

Figure S6. CV curves of (a) 4FSA-MOF, (b) Bim4FSA-MOF-3, (c) B-150, (d) B-250, (e) B-450, and (f) B-550 in 0.1 M NaOH solution at various concentrations.

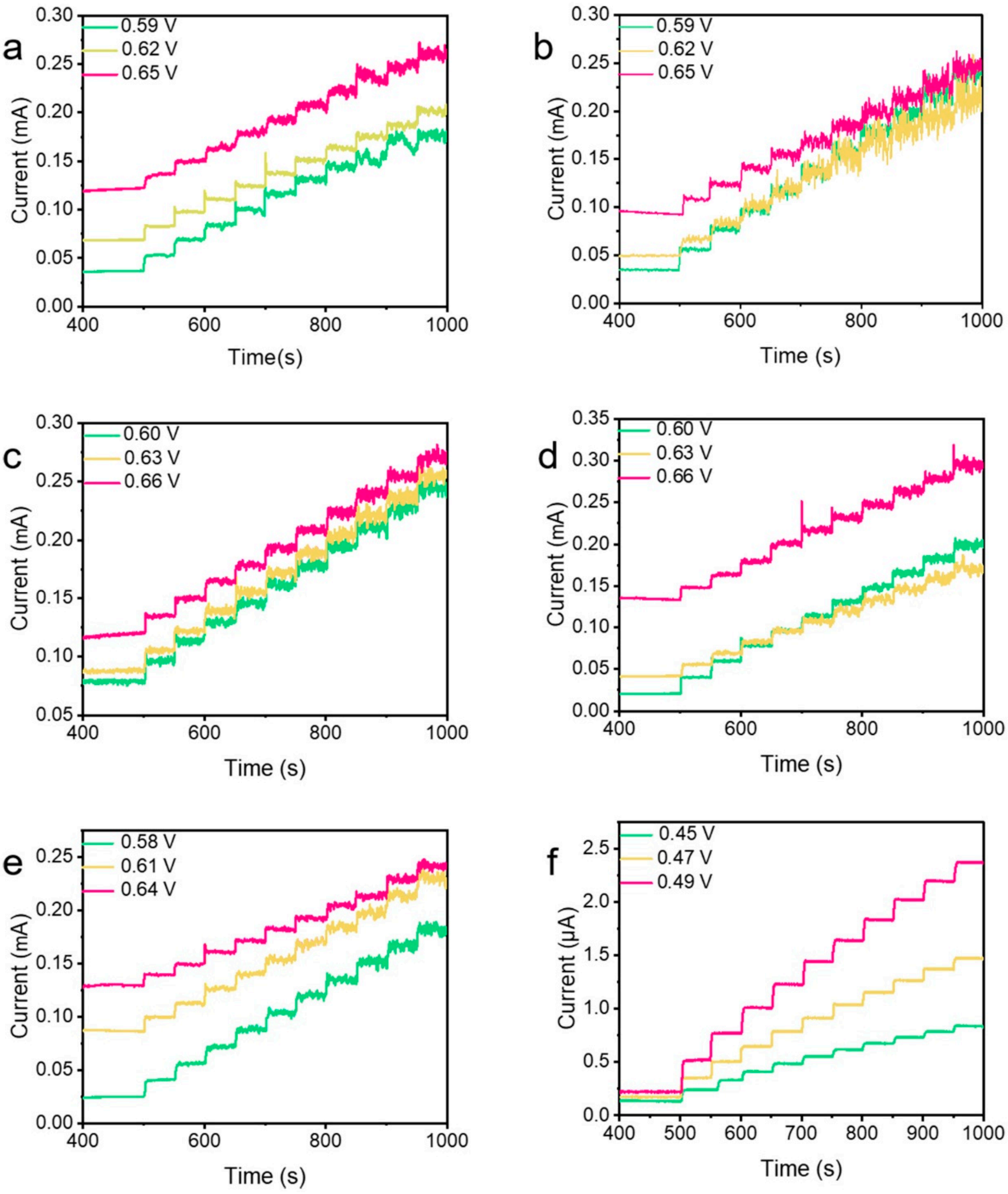

Figure S7. I-T curves of (a) 4FSA-MOF, (b) Bim4FSA-MOF-3, (c) B-150, (d) B-250, (e) B-450, and (f) B-550 recorded in 0.1 M NaOH with successive additions of 10 μL of 0.1 M glucose at 50 s intervals under different applied potentials.

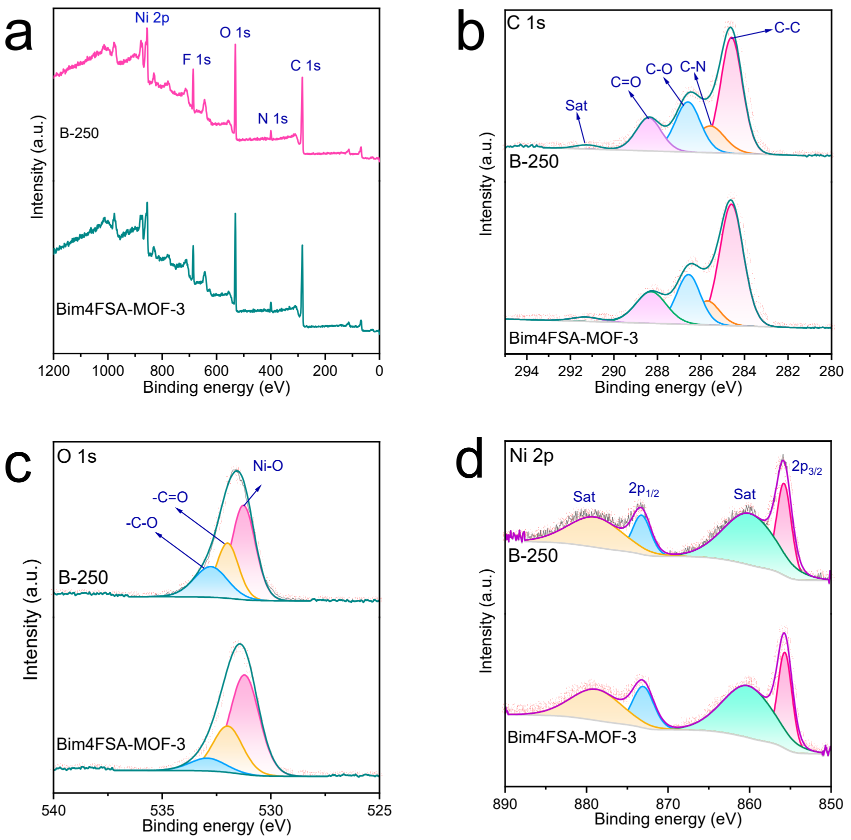

Figure S8. (a) XPS survey spectra, (b) high-resolution C 1s, (c) O 1s, and (d) Ni 2p XPS spectra of the Bim4FSA MOF-3 and B-250 samples.

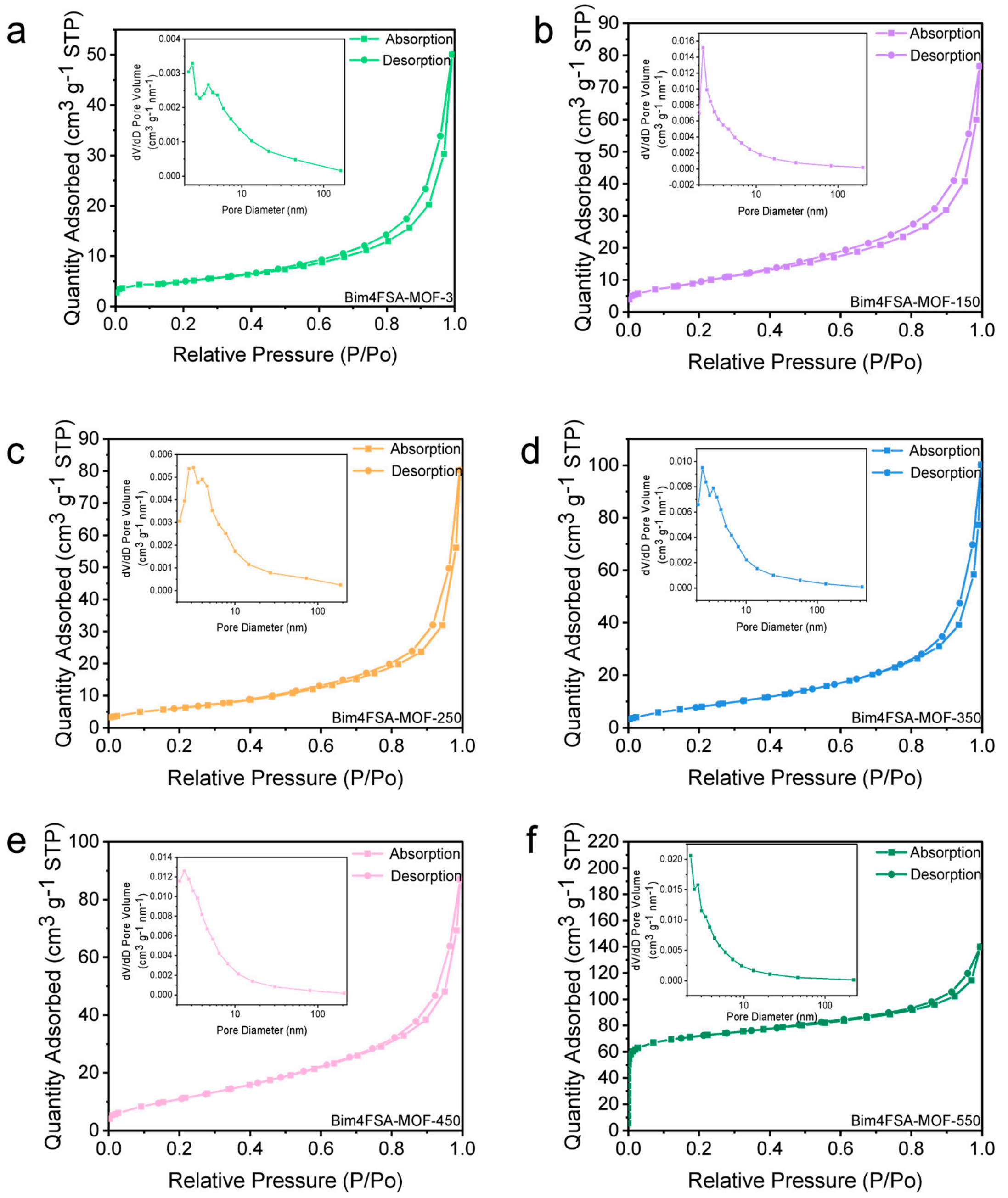

Figure S9. (a-f) The nitrogen adsorption-stripping isothermal line (illustrations for pore size distribution curve) of Bim4FSA-MOF-3, Bim4FSA-MOF-150, Bim4FSA-MOF-250, Bim4FSA-MOF-350, Bim4FSA-MOF-450 and Bim4FSA-MOF-550.

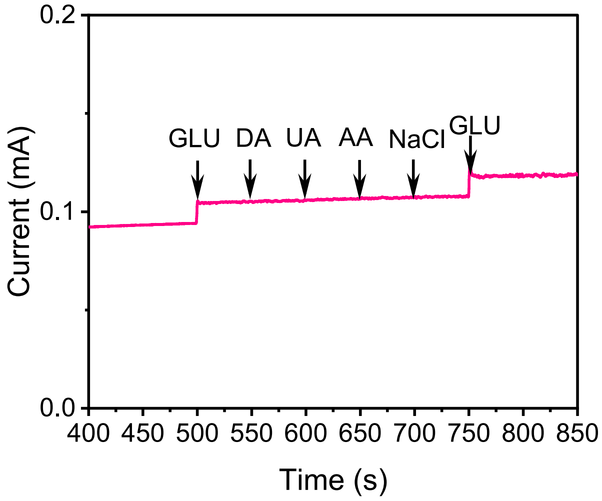

Figure S10. B-250 interference test curve.

Table S1. Pore size distribution of samples heat-treated at different temperatures

Samples	Pore diameter (nm)
Bim4FSA-MOF-3	12.03
B-150	17.07
B-250	20.13
B-350	15.39
B-450	11.48
B-550	3.66

Table S2. Comparison of other nickel-based MOFs and non-enzymatic glucose sensors

**Electrode Sample**	**linearity range** (μM)	**detection limit** (μM)	**Sensitivity** (μA mM^{-1} cm^{-2})	Ref.
Ni-MOF	0.5-2665.5	-	139.82	43
Ni_3C/Ni	1-6.5	0.280	299.40	44
Ni/NCNs-500	0.1-533.6	0.070	337.32	45
Ni-MOF/CNTs	1 -1600	0.820	1385.00	46
PNMOF-3	0.5-2665.5	1.640	907.54	47
B-250	0.5-2665.5	0.022	2986.45	our work
